# Carcinoma Cuniculatum of the Back: Dermoscopic and Ultrasonographic Findings

**DOI:** 10.7759/cureus.113936

**Published:** 2026-08-04

**Authors:** Manami Sugiyama, Natsuko Saito-Sasaki, Yu Sawada

**Affiliations:** 1 Dermatology, University of Occupational and Environmental Health Japan, Kitakyushu, JPN

**Keywords:** back, carcinoma cuniculatum, case report, dermoscopy, squamous cell carcinoma

## Abstract

Carcinoma cuniculatum (CC) is a rare, well-differentiated variant of cutaneous squamous cell carcinoma that is frequently misdiagnosed because of its deceptively benign clinical appearance. We report an unusual case of CC arising on the back that initially mimicked an epidermal cyst. A 55-year-old Japanese woman presented with a slowly enlarging subcutaneous nodule on her upper back that had been present for three years. Physical examination suggested an epidermal cyst, whereas ultrasonography demonstrated atypical findings that prompted excisional biopsy. However, the lesion rapidly enlarged within one month, ulcerated, and became a pedunculated tumor. Histopathological examination of an incisional biopsy revealed well-differentiated squamous cell carcinoma. Wide local excision was subsequently performed. Examination of the resected specimen demonstrated characteristic deeply penetrating keratin-filled branching sinus tracts with minimal cytological atypia, establishing the diagnosis of CC. This case emphasizes that rapidly progressive changes in lesions initially diagnosed as epidermal cysts should prompt reconsideration of the diagnosis and timely histopathological evaluation.

## Introduction

Carcinoma cuniculatum (CC) is a rare, well-differentiated variant of squamous cell carcinoma characterized by locally aggressive growth despite minimal cytological atypia [1,2]. Cutaneous CC accounts for fewer than 1% of cutaneous squamous cell carcinomas and occurs predominantly on the plantar surface of the foot [2]. Owing to its indolent clinical course and deceptively benign appearance, CC is frequently misdiagnosed as benign lesions, resulting in delayed diagnosis [3].

CC arising at non-plantar sites is exceptionally uncommon and may present an even greater diagnostic difficulty because clinicians are less likely to suspect the diagnosis outside its typical plantar location [4]. Herein, we report a case of CC arising on the back that initially mimicked an epidermal cyst. This case highlights the importance of recognizing rapid clinical progression and integrating clinical, imaging, and histopathological findings to avoid delayed diagnosis. In addition to the characteristic histopathological findings, this case provides detailed dermoscopic and ultrasonographic features that may facilitate earlier recognition of this rare entity.

## Case presentation

**Figure 1:**
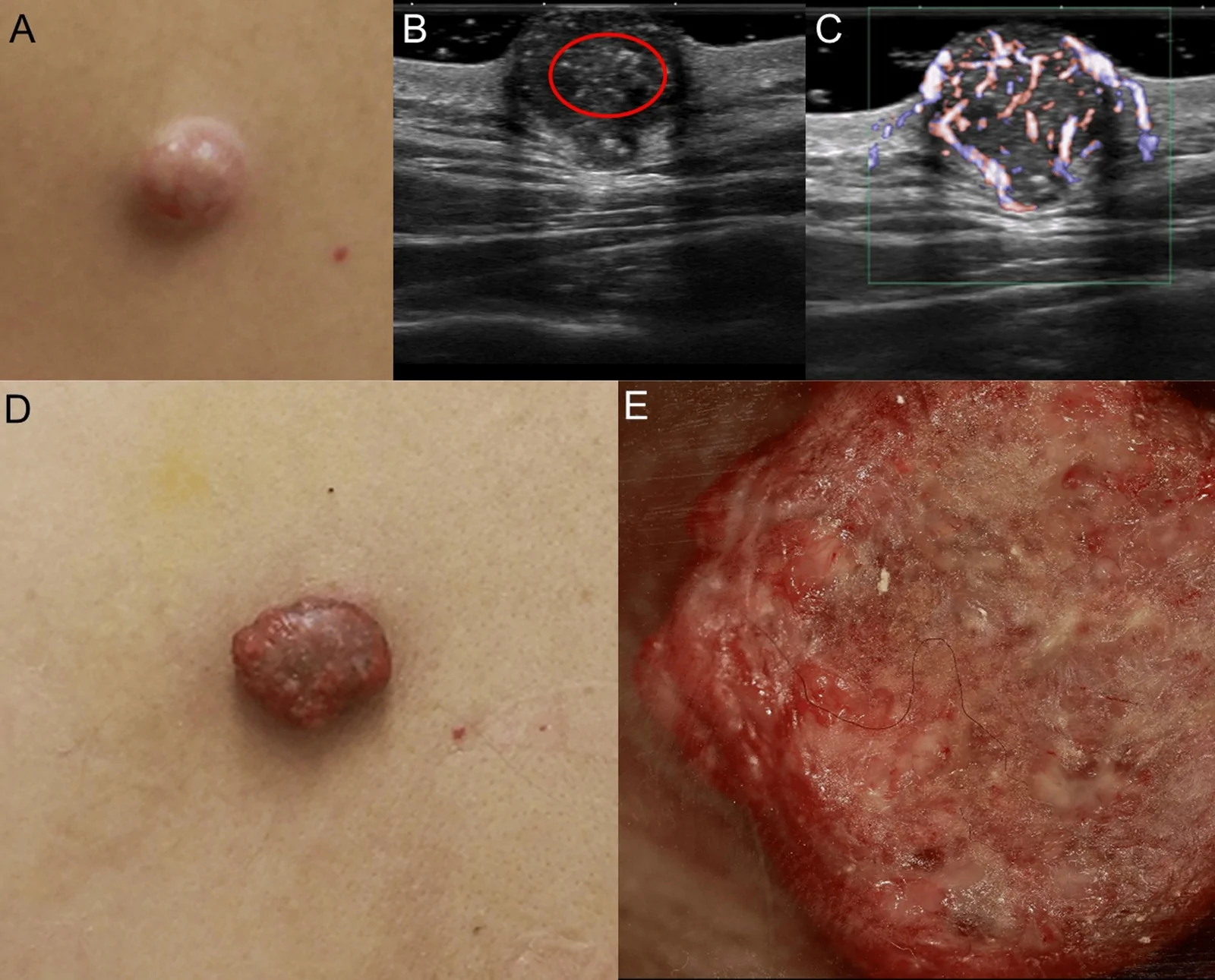
Clinical, ultrasonographic, and dermoscopic findings. (A) Initial clinical presentation showing a well-circumscribed, dome-shaped subcutaneous nodule on the upper back. (B) Gray-scale ultrasonography demonstrating a well-defined predominantly hypoechoic lesion containing multiple punctate hyperechoic foci (red circle) with posterior acoustic enhancement. (C) Color Doppler ultrasonography revealing abundant intralesional vascularity. (D) One month later, the lesion showed rapid enlargement and ulceration, forming a pedunculated erythematous tumor. (E) Dermoscopy demonstrating telangiectatic vessels and focal gray-whitish structureless areas.

A 55-year-old Japanese female first presented to our department in December 2025 for evaluation of a slowly enlarging subcutaneous mass on her upper back. She had first noticed the lesion approximately three years earlier, and it had gradually increased in size without pain. She had no history of immunosuppression, radiation exposure, trauma, or previous cutaneous malignancy. Physical examination revealed a well-circumscribed, dome-shaped dermal-to-subcutaneous nodule measuring 15 mm in diameter on the upper back (Figure 1A). The lesion was elastic-soft, freely mobile over the underlying tissue, and covered by mildly atrophic skin with superficial telangiectasia. There was no ulceration, tenderness, or discharge. No palpable regional lymphadenopathy was detected. High-frequency ultrasonography demonstrated a well-defined hypoechoic lesion measuring 15 × 13 × 15 mm, containing multiple punctate hyperechoic foci with posterior acoustic enhancement (Figure 1B). Color Doppler imaging revealed abundant intralesional vascularity (Figure 1C). Based on the clinical findings, the lesion was initially considered to be an epidermal cyst. However, ultrasonography demonstrated a predominantly solid hypoechoic lesion with intralesional vascularity rather than typical cystic features, raising concern that the lesion might not represent a simple epidermal cyst. Therefore, excisional biopsy was planned for definitive diagnosis.

Approximately one month after the initial consultation, the lesion enlarged rapidly and became ulcerated, forming a pedunculated erythematous tumor with exuberant granulation tissue (Figure 1D). Because of this unexpected clinical progression, an incisional biopsy was performed. Dermoscopic examination was performed using a CASIO Dermocamera DZ-S50 (CASIO Computer Co., Ltd., Tokyo, Japan) in polarized mode. Dermoscopy revealed telangiectatic vessels and focal gray-whitish structureless areas (Figure 1E). Dermoscopically, the lesion exhibited telangiectatic vessels and gray-whitish structureless areas. To our knowledge, dermoscopic findings of cutaneous CC have rarely been reported. The gray-whitish structureless areas may correspond to the marked keratinization of the tumor, whereas the telangiectatic vessels may reflect increased vascularity associated with the tumor. Ultrasonography demonstrated a predominantly solid hypoechoic lesion containing multiple punctate hyperechoic foci with posterior acoustic enhancement and intralesional vascularity. These findings may reflect the characteristic histopathological architecture of CC, including abundant keratin-filled sinus tracts within a predominantly solid epithelial proliferation. Collectively, these observations suggest that multimodal imaging may facilitate the diagnosis of CC.

Histopathological examination revealed proliferation of well-differentiated atypical squamous epithelium with marked keratinization, leading to a diagnosis of well-differentiated squamous cell carcinoma. Contrast-enhanced computed tomography demonstrated no evidence of deep muscle invasion, regional lymph node involvement, or distant metastasis.

**Figure 2:**
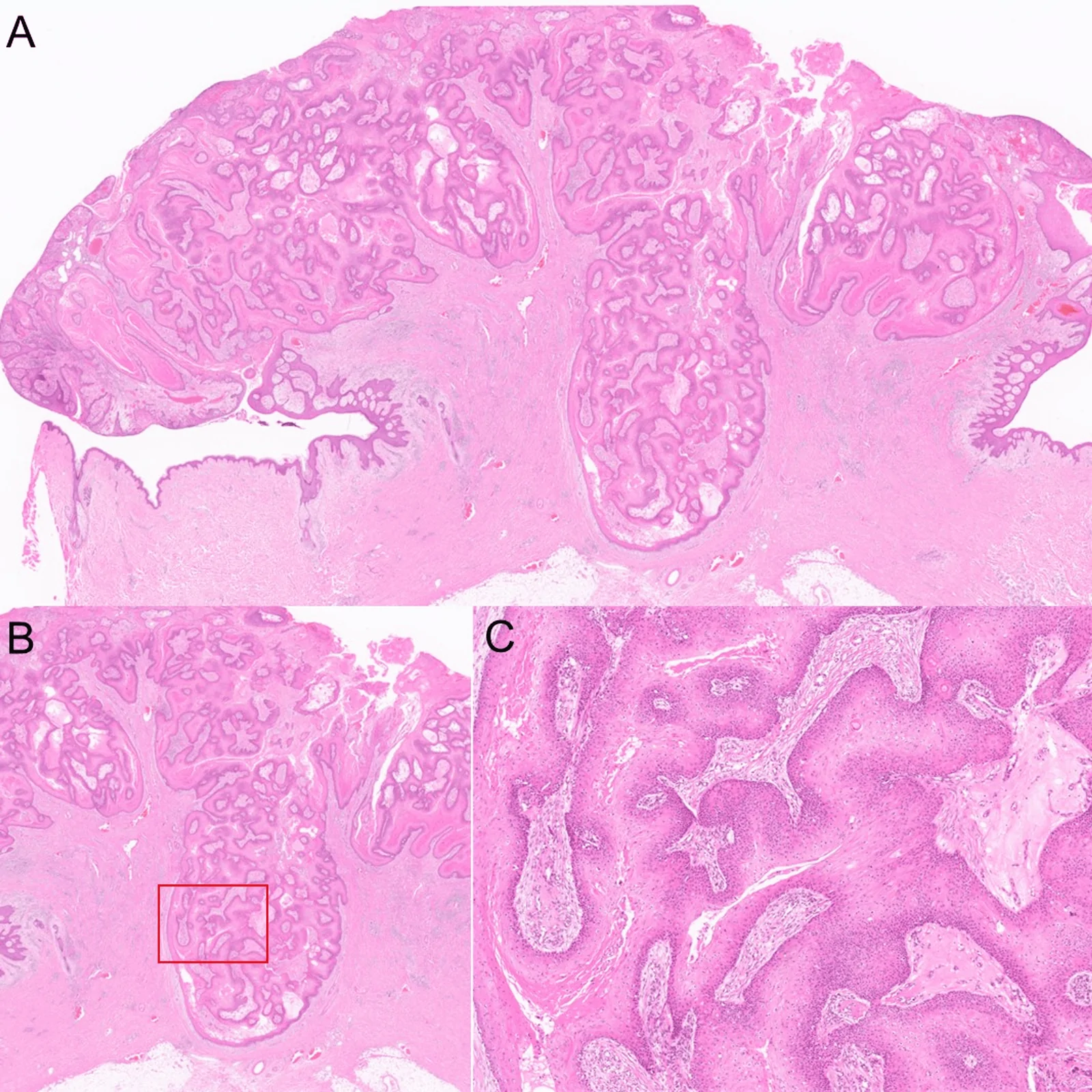
Histopathological findings. (A) Low-power view showing a well-circumscribed endophytic tumor composed of characteristic deeply penetrating keratin-filled branching sinus tracts extending into the dermis. (B) Intermediate-power view demonstrating the characteristic branching keratin-filled sinus tracts. (C) Higher-magnification view of the boxed area in (B), showing abundant keratinization with minimal cytological atypia.

Wide local excision was subsequently performed with a 6-mm surgical margin. Histopathological examination of the resected specimen demonstrated a well-circumscribed endophytic tumor composed of deeply penetrating, branching keratin-filled sinus tracts extending into the dermis (Figures 2A-2C). The tumor exhibited abundant keratinization with only mild cytological atypia, findings characteristic of CC. Both the peripheral and deep surgical margins were histologically negative. The postoperative course was uneventful, and no local recurrence or metastasis has been observed during six months of follow-up.

## Discussion

The present case illustrates the diagnostic difficulty of CC because the lesion clinically mimicked an epidermal cyst despite atypical ultrasonographic findings. This difficulty was further compounded by its unusual location on the back, where cutaneous CC is exceptionally rare [5,6]. Previous reports have shown that cutaneous CC occurs predominantly on the plantar surface, with only a few cases reported on the trunk [2,5,6]. Similar to those cases, our patient presented with a slowly enlarging lesion that was initially considered benign. However, unlike most reported cases, the lesion in our patient enlarged rapidly within one month and ulcerated, leading to prompt histopathological evaluation. Doppler ultrasonography demonstrated intralesional vascularity, which may have represented an imaging clue to the underlying malignancy. These findings suggest that rapidly changing lesions, particularly those with atypical imaging features, warrant careful reassessment even when the initial clinical impression is benign.

Histopathologically, CC is characterized by deeply penetrating keratin-filled sinus tracts with only minimal cytological atypia [2]. These histopathological findings are consistent with those reported in previous case series, in which deeply penetrating keratin-filled sinus tracts with minimal cytological atypia are considered the hallmark of CC [2,5]. Because the superficial portion of the lesion often resembles pseudoepitheliomatous hyperplasia or a keratinous cyst, superficial biopsy specimens may fail to demonstrate the characteristic architecture. Consequently, adequate tissue sampling including the deep advancing front is essential for establishing the diagnosis. In the present case, examination of the entire excised specimen was crucial for the definitive diagnosis. This distinctive growth pattern has been attributed, at least in part, to preserved expression of cell adhesion molecules such as laminin-5 γ2, integrin α6, and E-cadherin, which are thought to maintain cohesive downward tumor growth [7].

Complete surgical excision remains the treatment of choice because CC is locally invasive despite its relatively low metastatic potential. Adequate surgical margins are important to minimize local recurrence, whereas lymph node or distant metastases are distinctly uncommon [3,8]. Consistent with previous reports, our patient underwent complete excision with histologically negative margins and has remained free of recurrence during the six-month follow-up [3,8].

Although no local recurrence or metastasis has been observed during the six-month follow-up period, the relatively short duration of follow-up is a limitation of this report, and continued long-term surveillance is necessary to better assess the risk of recurrence.

## Conclusions

CC is a rare, well-differentiated variant of squamous cell carcinoma that is frequently misdiagnosed because of its slow-growing clinical course and benign-appearing features. Dermoscopy and ultrasonography may provide valuable complementary information for preoperative assessment, while histopathological examination remains essential for a definitive diagnosis. Early recognition and complete surgical excision are crucial to achieve local disease control and prevent recurrence.
